# Microtubule Targeting Agents in Disease: Classic Drugs, Novel Roles

**DOI:** 10.3390/cancers13225650

**Published:** 2021-11-12

**Authors:** Linda Wordeman, Juan Jesus Vicente

**Affiliations:** Department of Physiology and Biophysics, University of Washington School of Medicine, Seattle, WA 98195, USA; worde@uw.edu

**Keywords:** microtubules (MTs), microtubule-targeting agent (MTA), pathogen, tauopathies, cancer, migration, vascular formation

## Abstract

**Simple Summary:**

Microtubules (MTs) are highly conserved proteins present in all eukaryotic organisms. They form the cell cytoskeleton, and its function is essential for a large number of biological processes. Drugs against MTs (i.e., microtubule-targeting agents or MTAs) have been used for centuries to treat arthritis and gout. In the last 100 years, new MTAs either isolated from natural sources or synthesized in labs have been used to treat a great variety of human illnesses, from cancer and neurodegenerative diseases to the elimination of parasites. In this review, we analyze how MTAs bind to MTs, and the molecular mechanisms behind MTAs function, and we describe the last and novel roles of these drugs.

**Abstract:**

Microtubule-targeting agents (MTAs) represent one of the most successful first-line therapies prescribed for cancer treatment. They interfere with microtubule (MT) dynamics by either stabilizing or destabilizing MTs, and in culture, they are believed to kill cells via apoptosis after eliciting mitotic arrest, among other mechanisms. This classical view of MTA therapies persisted for many years. However, the limited success of drugs specifically targeting mitotic proteins, and the slow growing rate of most human tumors forces a reevaluation of the mechanism of action of MTAs. Studies from the last decade suggest that the killing efficiency of MTAs arises from a combination of interphase and mitotic effects. Moreover, MTs have also been implicated in other therapeutically relevant activities, such as decreasing angiogenesis, blocking cell migration, reducing metastasis, and activating innate immunity to promote proinflammatory responses. Two key problems associated with MTA therapy are acquired drug resistance and systemic toxicity. Accordingly, novel and effective MTAs are being designed with an eye toward reducing toxicity without compromising efficacy or promoting resistance. Here, we will review the mechanism of action of MTAs, the signaling pathways they affect, their impact on cancer and other illnesses, and the promising new therapeutic applications of these classic drugs.

## 1. Microtubules and Tubulin

**Figure 1:**
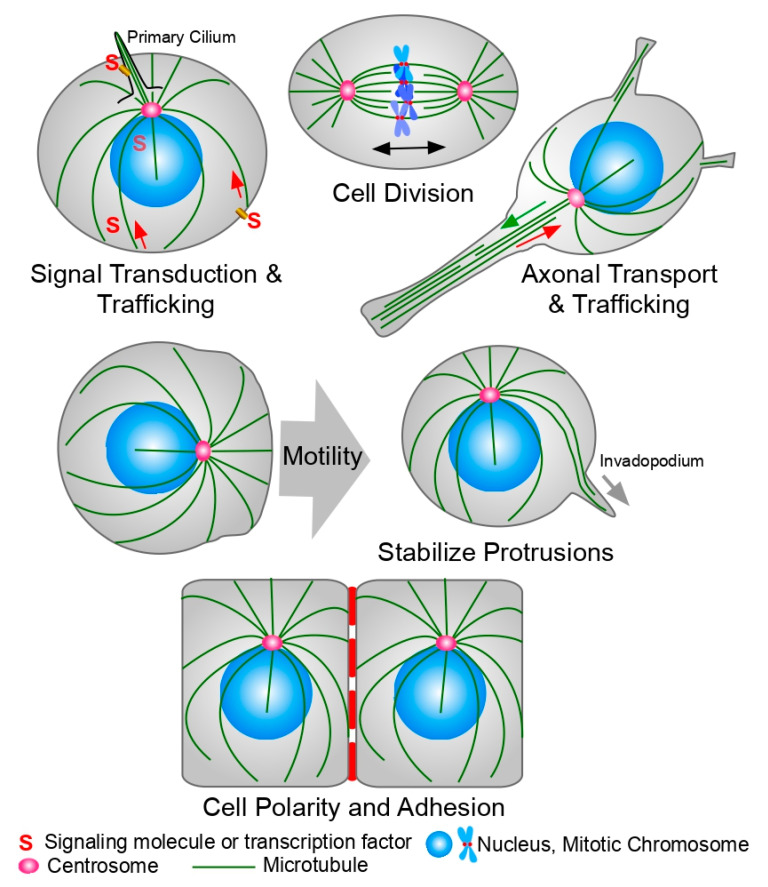
Microtubules (MTs) functions in the cell. MTs play roles in a variety of cellular functions. **Top row, left:** MTs are involved in the transport of a variety of molecules, vesicles, and organelles inside the cell, affecting signaling transduction pathways. They are also essential in the formation and maintenance of the primary cilium, a signaling hub for the cell. **Top row, center:** MTs form the mitotic spindle, the machinery in charge of chromosome segregation during mitosis. **Top row, right:** MTs form the tracks used by molecular motors to transport neurotransmitters through the axon to the synaptic cleft. **Center row:** Proper organization of the MT cytoskeleton and centrosome localization is important to stabilize the migrating leading edge, for cell motility and for the formation of the invadopodium, a cellular extension of the cell membrane required for invasion of adjacent tissues. **Bottom row:** MTs are also important during the adhesion and the basal-to-lumen cell polarity establishment of epithelial cells. They also play an important role during the formation and turnover of focal adhesions. See the text for details.

Microtubules (MTs) are hollow cylindrical polymers composed of tubulin dimers. They play vital roles in a wide variety of cellular functions: intracellular trafficking, cell shape and morphology, positioning of organelles inside the cell, cell motility and migration, and the assembly of the mitotic spindle, which is the machinery responsible for chromosome segregation during mitosis [1] (Figure 1). Tubulin is a 50 kDa globular GTP-binding protein that is found in all eukaryotic cells. There are six members of the tubulin family in eukaryotic cells: alpha (α), beta (β), gamma (ɣ), delta (δ), epsilon (ε), and zeta (ζ) [1,2]. However, not all organisms possess all the tubulin genes. For example, zeta tubulin is not present in humans. The widely distributed α and β tubulins assemble to form heterodimers that will ultimately constitute the cytoplasmic microtubules, and they have been found in all eukaryotic cells studied thus far. Both of them bind GTP, but only β tubulin is able to hydrolyze GTP during microtubule polymerization. ɣ tubulin is part of the machinery that nucleates microtubule growth. δ, ε, and ζ tubulins are part of cilia, flagella, and basal bodies, and they are generally specific to organisms that possess these structures. In humans, there are 23 functional genes coding for tubulin [2], with α and β constituting the elementary building blocks of MTs and ɣ, δ, and ε localizing to the centrosome. The α-β tubulin heterodimers polymerize into a linear protofilament, which associates laterally with more protofilaments to form the pseudo-helical structure that is the MT. Most MTs are composed of 13 protofilaments, but MTs with different numbers of protofilaments have been observed in different species [3].

MTs present with a polarity consisting of a plus-end where tubulin dimers are preferentially added, and a minus-end often embedded within the centrosome, the chief microtubule organizing center (MTOC) of most human cells. Polarity emerges due to the head-to-tail orientation of the α-β tubulin heterodimers inside the MT lattice, with the α subunit pointing towards the minus-end of the MT and the β subunit towards the plus-end. Of the two, the β-tubulin subunit hydrolyzes GTP after the incorporation of the tubulin dimer to the microtubule [4]. Prior to this hydrolysis event, GTP-β-tubulin forms a structural cap that stabilizes the MT. As long as there is enough GTP-β-tubulin in the vicinity of the MT tip, more dimers will continue to be added to the tip and the MT will continue growing. However, if the growth rate falls below the rate of GTP-β-tubulin hydrolysis, loss of the stabilizing cap triggers the disassembly of the MT in a process called MT catastrophe. Once a new cap is formed, the MT can begin growing again. Periods of growth and shrinking alternate in a cycle known as dynamic instability [5]. In cells, an ample cohort of microtubule-associated proteins like the end-binding proteins EB1, EB2, and EB3 regulate this process [6,7,8]. This dynamic turnover of MT polymer is essential for virtually all MT-dependent biological processes.

## 2. Microtubule-Targeting Agents (MTAs)

**Figure 2:**
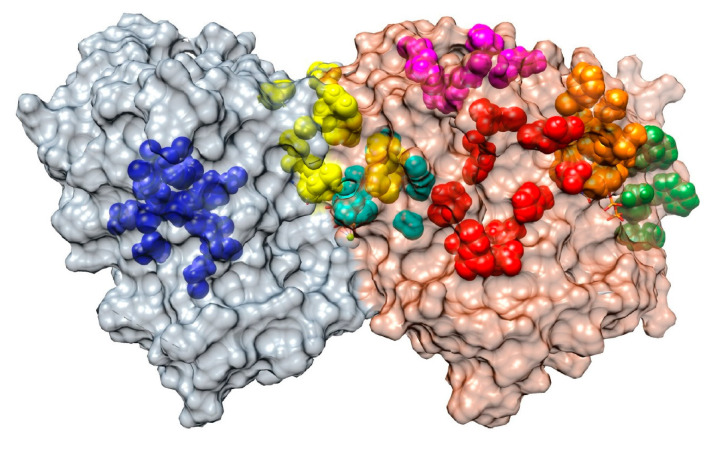
Microtubule-targeting agents (MTAs) biding sites on the tubulin dimer. The αβ-tubulin dimer is shown in gray and light brown with the α subunit on the left and the β subunit on the right. Colored representative structures of the ligands are located on their binding sites. Color code: blue, Pironetin; yellow, Gatorbulin; cyan, colchicine; red, taxane; orange, vinca; magenta, laulimalide/peloruside; green, Maytansine. See text for details.

MTAs are a large and diverse family of chemical compounds that are able to bind tubulin and interfere with the dynamic behavior of MTs, either by stabilizing or destabilizing the MT polymer. Accordingly, the simplest classification of these drugs is into two groups: MT-stabilizing agents (MSAs) and MT-destabilizing agents (MDAs). All MTAs bind to one of these seven sites in the tubulin dimers [9,10,11] (Figure 2).

− Vinca domain (MDA, orange in Figure 2): this domain is located in the β tubulin monomer at the inter-dimer interface between two longitudinally aligned tubulin dimers. Drugs that bind to this domain inhibit the assembly of tubulin by sequestering tubulin into paracrystalline aggregates. Examples of these drugs are vincristine, vinblastine, and vindesine [12,13,14,15].
− Colchicine site (MDA, cyan in Figure 2): this site is in the β tubulin monomer in a deep pocket space between the αβ-tubulin dimer itself. Binding to this site inhibits microtubule formation by preventing a conformational change in tubulin required for polymerization. Colchicine, benzimidazoles (e.g., nocodazole), and combretastatins are among the drugs that bind to this site [16,17].
− Taxane site (MSA, red in Figure 2): it is located at the β tubulin monomer in the lumen of microtubules. Binding to this site stabilizes the MT lattice. Interestingly, MT-stabilizing drugs, such as paclitaxel and epothilone, achieve their microtubule-stabilizing effect through pharmacologically distinct mechanisms despite binding competitively to the same taxane site [18].
− Maytansine domain (MDA, green in Figure 2): it is located close to the vinca site on an exposed pocket of β-tubulin. The binding to this site inhibits the addition of further tubulin dimers to the plus ends. Maytansine and Spongistatin are examples of molecules binding to this site [19,20,21].
− Laulimalide/peloruside site (MSA, magenta in Figure 2): this site is located in a pocket of the β-tubulin that faces the outside of the MT. Studies suggest that they inhibit microtubule disassembly by acting as molecular ‘clamps’ that hold together protofilaments. The drugs Laulimalide and Peloruside isolated from marine sponges gave name to this group [22,23,24].
− Pironetin site (MDA, blue in Figure 2): it targets the α-tubulin subunit and destabilizes MTs by inhibiting longitudinal tubulin–tubulin interactions and preventing the incorporation of new heterodimers to the MT tip [25,26,27,28].
− Gatorbulin site (MDA, yellow in Figure 2): this new site has been described in 2021, is located in the α-tubulin subunit (between α- and β-tubulin) close to the colchicine site, and it functions in a similar way to the vinca site, creating a wedge between two longitudinally aligned tubulin dimers at the end of MTs [10]. Recently, a new compound called Cevipabulin has been described to also bind to this pocket [11].

For an excellent in-depth review of MTA binding sites and their mechanism of action, see Steinmetz and Prota in 2018 [9]. MSAs and MDAs exhibit opposite effects on MT polymer mass at high concentrations, inducing either depolymerization or stabilization of the MTs, and changing the ratio of monomer and polymer. At low concentrations, however, they can influence MT dynamics to affect cellular functions without appreciable changes in the total amount of MT polymer. Drugs binding to the vinca, colchicine, and taxane sites comprise the most well-studied MTAs, and they have been used as medicines for a long time. Colchicine, for example, originally extracted from the plant autumn crocus (*Colchicum autumnale*), is probably the oldest and first reported MTA used as a therapeutic drug. The Egyptian medical text known as the “Ebers papyrus”, dated from 1500 BC, described the use of the plant autumn crocus as a remedy for inflammation and rheumatism [29]. Colchicine has also been used for centuries to treat gout [29,30]. However, in the last century, MTAs have been also used as herbicides, anti-parasitics and anti-fungal agents, to treat neurodegenerative disease and for cancer treatment.

## 3. MTAs and Pathogens

Tubulin is conserved among all eukaryotic cells, from *Homo sapiens* to the deeply rooted supergroup Excavata [2]. However, there are differences in the tubulin sequence between humans, parasites, and fungi that make this protein an appropriate target for antifungal and antiparasitic drugs [31,32,33]. There are, in fact, several MTAs used as antiparasitic drugs that bind more efficiently to pathogen tubulin than to human tubulin [34].

Benzimidazole is an aromatic organic compound resulting from the fusion of imidazole and benzene. Through modifications of the benzimidazole group, chemists have generated a vast group of compounds that have been used successfully to affect a variety of biological activities. It has been shown that compounds derived from benzimidazole have antitumor activity, antiviral activity, and anti-inflammatory, antihypertensive, and anticoagulant properties [35]. A group of benzimidazole compounds that bind close to the colchicine binding site of β-tubulin have been used as antihelminthics to treat a great variety of parasitic worm infections. Examples of these compounds are albendazole, mebendazole, triclabendazole, and thiabendazole. In vitro studies of benzimidazole compounds show that they bind with greater affinity to the β-tubulin site of worms than the equivalent site in humans, effectively blocking MT polymerization at lower drug concentrations in the parasites [36]. However, the therapeutic efficiency of benzimidazoles is based on their poor solubility in water and poor absorption in the intestines. For example, the absorption rate for mebendazole by the human intestine is between 1 and 5% unless it is administered with high-fat foods. Thus, mebendazole affects the parasite in the human intestine, sparing the host cells [37,38,39,40]. Benzimidazole compounds carbendazim, benomyl, thiophanate-methyl, and thiabendazole have also been used as fungicides based, also, on their lack of a measurable interaction with mammalian tubulin [41,42,43].

MTAs have also been used in agriculture for weed control [44]. Dinitroanilines are a class of chemicals derived from either dinitrobenzene or aniline that are used as dyes, pesticides, and herbicides. They are also used as antiparasitic drugs as they bind to α-tubulin in parasites, while exhibiting a lack of measurable affinity for vertebrate α-tubulin [45,46,47,48,49]. Among these compounds, we have trifluralin (herbicide and antiparasitic) [47] and oryzalin (herbicide and antiparasitic) [50].

A couple of excellent reviews about MTAs as antifungal and antiparasitic drugs were published in 2008 and 2011 [34,51].

## 4. MTAs and Neurodegenerative Diseases

The brain is critically dependent on MTs for function. Neurons possess long axons full of parallel MTs that act as tracks for the transport of neurotransmitter from the cell body to the synaptic cleft (Figure 1). This transport is mediated by kinesins and dyneins, which serve as MT-dependent molecular motors [52]. In most neurodegenerative diseases, neuronal MTs are disrupted and the transport of neurotransmitters via MTs in the axon is impaired. Thus, MSAs have the potential to treat the symptoms of neurodegenerative diseases like Parkinson’s and Alzheimer’s, protecting the MTs in the axons and restoring synaptic transmission [53,54,55,56,57,58,59].

### Tauopathies

Tau proteins are a group of microtubule-associated proteins that originate by alternative splicing from the MAPT gene in the chromosome 17 in humans. There are six isoforms expressed in the human adult brain, where they play an essential role stabilizing the MTs in the axons of neurons and are involved in neural transport and axonal growth. Defects in the phosphorylation levels of Tau proteins lead to the formation of fibrillar inclusions composed of hyperphosphorylated tau protein [60,61,62]. Neurodegenerative disorders affecting Tau proteins are called tauopathies, with Alzheimer’s and Parkinson’s diseases being the most well-known and most studied [63,64].

Alzheimer’s disease is one of the most prevalent tauopathies in the world. In Alzheimer’s disease, a hyper-phosphorylated form of tau self-assembles into polymers to create insoluble deposits in the neurons. The creation of these aggregates decreases the ability of tau to bind and stabilize MTs, resulting in axonal and neural dysfunction [65,66]. Alzheimer’s disease is also characterized by the accumulation of β-amyloid plaques in the brain, and most of the research in Alzheimer’s disease has been focused on decreasing the levels of amyloid-β (Aβ) peptides in the brain. However, the lack of success using drugs to target the Aβ peptides changed the focus toward reducing the accumulation of Tau protein, and the development of molecules that are able to compensate the lack of normal Tau function [59,67]. Tau, like paclitaxel, binds to β-tubulin and stabilizes MT polymer. Thus, there exists a formal possibility that MSAs could stabilize MTs in situations where hyperphosphorylated aggregations of Tau cannot properly exert their function [53,56,59,68,69,70]. In support of this idea, treatment of a tau transgenic mouse model (mice with a pre-existing tau pathology) with paclitaxel restored fast axonal transport in spinal axons and improved motor impairment. At the cellular level, it was seen an increase in MT number and stable (detyrosinated) MTs [71]. The MT-stabilizing triazolopyrimidine CNDR-51657 significantly lowered tau pathology and improved cognitive function in transgenic mouse models of tauopathy [72]. One of the drugs that has been especially successful in treating tau transgenic mouse models is the MSA epothilone [73]. Paclitaxel and derivatives cannot cross the blood–brain barrier (BBB). Epothilone D was identified as a brain penetrant MSA, and when used at even lower doses than the ones required for cancer treatment, it was able to improve the axonal MT density and transport, decrease axonal dystrophy, and improve the cognitive deficits in a transgenic Tau mouse model [56,74]. These improvements appear to be related to the recovery of normal MT dynamics. In a different study, two different tau transgenic mouse models exhibited age-dependent increases in MT dynamics that were dependent on tau expression levels. Treatment of these mice with epothilone returned MT dynamics levels to basal levels, and had beneficial effects on tau pathology and neurodegeneration [75]. In all these cases, the treatment used a low concentration of drugs and recorded no other adverse effects in the mice. A good review about Alzheimer’s disease, tau protein aggregation, and different therapeutic agents was published in 2017 [66].

There is growing evidence that MT alteration is also a component of Parkinson’s disease. MT defects induced with 1-methyl-4-phenyl-1,2,3,6-tetrahydropyridine (MPTP) in mice were rescued with Epothilone D treatment, suggesting that MSAs could be employed in the treatment of Parkinson’s disease [55,76].

MSAs may also improve axonal growth and recovery after a stroke in mouse models. The use of Epothilone increases the number and length of neural projections from peri-infarct tissue, and improves forelimb motor function [77]. Epothilone B also promotes axon regeneration and fibroblast migration after spinal cord injury [78].

Therefore, MSAs may constitute a treatment option for neurodegenerative illness. However, there are two drawbacks when using MSAs to treat the nervous system. The first one is peripheral neurotoxicity. MTAs disrupt the MT network in the axon, which allows kinesin molecular motors to transport organelles and vesicles with neurotransmitters to the synaptic cleft, leading to muscle pain and weakness, numbness, and sensory defects [79,80,81]. To address this problem, new taxanes formulations have been developed to improve delivery and reduce side effects [82,83]. For example, the epothilone B analogue ixabepilone has shown some potential to reduce peripheral neurotoxicity without affecting therapeutic capacity [84,85]. Different pharmacological strategies like the use of antidepressants and anticonvulsants, and the use of glutathione, glutamine, and amifostine have also been employed to treat neuropathy, but their benefits are limited [82,84,86]. As today, there are several approaches at different clinical trial phases to address the neuropathy problem related to MTAs [86]. Nevertheless, dose reduction and treatment delay are the most used management tools for MTAs-induced neuropathy [84]. The second drawback is that most of MSAs are not able to cross the BBB as the brain tissue contains a high number of P-glycoprotein drug efflux pumps to remove most chemicals [87]. While paclitaxel and its derivates cannot cross the BBB, Sagopilone and other Epothilone derivates are able to cross the BBB [76,88].

## 5. MTAs and Cancer

Cancer is the second leading cause of death in the world [89]. The last decade has seen almost 600,000 deaths per year just in the US [90]. Accordingly, MTAs have been used since the mid-1960s as a first-line therapy for cancer treatment [91,92,93,94,95]. While vinca alkaloid and taxane compounds are broadly used for cancer treatment, colchicine use has been limited to the treatment of gout due to its toxicity [30,96,97]. It is challenging to achieve a safe dose of colchicine, and unintentional toxicity can lead to multiorgan failure and death [98]. Despite having different binding sites, the majority of MTAs share a similar mechanism: they affect MT dynamics by increasing, decreasing, or pausing MT polymerization rates. MTs play essential roles in most cell physiological events and the dynamic turnover of MTs is, in turn, essential to their proper function. Thus, altering MT dynamics usually has extremely deleterious effects on cells and often leads to cell death. The most obvious and well-studied defect in cells treated with MTAs is the disruption of the mitotic spindle. The mitotic spindle is composed of microtubules and it is the cell machinery responsible for the proper segregation of chromosomes during mitosis [99]. MTAs disrupt MT dynamics in the spindle, eliciting the mitotic checkpoint, arresting cells in metaphase, and triggering cell death [96]. This led to the conclusion that these drugs kill tumor cells while they are in mitosis. This spurred the development of new chemotherapeutic drugs specifically targeting mitotic proteins, especially mitotic kinases like Aurora and Polo kinases [100,101,102,103,104]. These mitotic-specific drugs have had some success, but they have never attained the tumor killing efficiency of MTAs. Furthermore, most human tumors exhibit slow doubling times, with a mitotic index between 1 and 3% at any given time that cannot account for the tumor shrinkage rate triggered by MTAs [105,106,107]. This demands further investigation of the mechanism of action of MTAs, as most probably they exert their function through a combination of mitotic and interphase effects [108]. Moreover, the possibility exists that the effect of MTAs during mitosis could even be detrimental for cancer treatment. Changes of MT dynamics caused by MTAs also alter the MT–chromosome attachments, which ultimately can lead to chromosome missegregation during anaphase. The increased rate of chromosome missegregation in mitosis is known as chromosome instability (CIN), and it is the cause of aneuploidy. CIN can drive tumorigenesis and cancer development, and has been correlated with drug resistance, therapy failure, metastasis, and poor prognosis (studied and reviewed in [109,110,111,112,113,114,115,116,117,118,119]). Interestingly, while moderate levels of CIN can promote tumorigenesis by promoting karyotype evolution toward the goal of increasing cell survival and growth, high levels of CIN often induced by MTAs kill tumor cells [120]. Importantly, arresting cells in mitosis can be a double-edged sword as these arrested cells can either die or exit mitosis without completing chromosome segregation or cytokinesis. Some of these cells will die in the subsequent cell cycle, but some are able to continue living as polyploid cells, becoming polyploid giant cells (PGCCs) [121,122,123,124,125]. These PGCCs are seen as cancer stem-like cells and reportedly exhibit increased metastasis, increased invasive phenotypes, increased migratory capabilities, and the expression of epithelial to mesenchymal transition markers [121,122,126]. Cells can manifest a wide variation in outcomes after treatment with different MTAs [124]. Even cells from the same cell line can present a variety of cell fates: dying in the first mitosis after MTA treatment, exiting mitosis to arrest in interphase, entering mitosis two or three times after treatment and then dying in interphase or mitosis, dying in interphase after the first mitotic arrest, and finally, dying in interphase without ever entering mitosis [124]. Moreover, the heterogeneity in the cell death in response to MTAs within the same cell line does not appear to be genetically predeterminated. Thus, there is a fine balance between MTAs’ effect on mitosis and cell fate. Clearly, MTAs can kill cells in mitosis and interphase; however, the molecular pathways behind the ability of these drugs to kill cells in interphase remain unclear.

### 5.1. How Can MTAs Kill Cancer Cells through Mitosis-Independent Mechanisms?

It is possible that MTAs are killing cells in interphase because they interfere with processes like vesicular transport, cell morphology and migration, and/or cell signaling pathways [106]. For example, MTAs are very effective in cancer treatment when used together with DNA-damaging agents or in conjunction with DNA damage induced by radiotherapy. This could be explained, in part, by the fact that MTs are essential for the transport of DNA repair proteins to the nucleus, and that MTAs interfere with and block this transport [127]. MTAs could also directly trigger apoptosis. For example, Taxanes kill cells through a different mechanism depending on the concentration. At high concentrations, they completely suppress MT dynamics, resulting in necrosis. However, at low concentrations, taxanes are able to trigger caspase-induced apoptosis [128]. Finally, MTAs may disturb the vascular tissue around the tumor that provides the cancer cells with oxygen and nutrients [129,130]. Independently of the precise mechanism triggered by MTAs, it appears that these drugs often affect cancer cells more severely than normal cells. Cancer cells walk a tightrope between proliferation, cell cycle arrest, genome instability, and unfavorable growth conditions [107,131,132]. It has been proposed that cancer cells operate very close to their cell death threshold at all times and that MTAs can disrupt this tenuous balance to specifically trigger cancer cell death.

### 5.2. MTAs and Intracellular Trafficking

MTs are the cellular tracks for the transport of vesicles, proteins, mRNAs, and organelles inside the cell. For example, the transport of proteins to the nucleus and the internalization of receptors requires MTs [133,134]. The tumor suppressor protein p53 requires both intact MTs and the molecular motor dynein to be transported to the nucleus. High doses of MTAs that completely disassemble the MT network will impede the transport of p53 to the nucleus. Paradoxically, it has been shown that subtly altering MT dynamics with low concentrations of MTAs can increase the amount of p53 inside the nucleus, which can sensitize cells to p53-dependent apoptotic cell death [135,136,137]. Another protein that requires MTs for the transport to the nucleus is the parathyroid hormone (PTH)-related protein [138]. Paclitaxel treatment also affects EGFR internalization and endocytic trafficking [139]. MTAs disturb hypoxia-inducible factor 1α (HIF-1α) protein trafficking and function, impairing tumor angiogenesis [140,141,142].

Taxanes are among the most successful drugs used as cancer treatments. Taxol (the commercial brand name of Paclitaxel) has been used for ovarian cancer since 1992 and breast cancer since 1994 [143,144,145]. Additionally, Docetaxel (commercially branded as Taxotere) is used for breast, lung, and prostate cancer [146]. In melanoma cells, paclitaxel reduces invasiveness, disrupting the transport and exocytosis of the metalloproteases necessary to break and degrade the extracellular matrix component [147]. Taxanes also represent the best agents for hormone-refractory prostate cancer as they prevent the transport of the androgen receptor to the nucleus, a necessary step for tumor progression [148,149]. Paclitaxel treatment of the prostate cancer cell line CRPC (castration-resistant prostate cancer) inhibits androgen receptor (AR) activity. Taxanes decreased the expression of androgen receptor (AR)-activated genes, such as prostate-specific antigen (PSA), and increases the expression of the AR repression gene maspin, resulting in global inhibition of AR activity. Paclitaxel also induces nuclear accumulation of the transcription factor FOXO1, a known AR suppressive nuclear factor [150,151].

Another example of the use of MTAs to disturb intracellular trafficking with therapeutic potential is the localization of the Hedgehog (Hh) signaling pathway components to the primary cilium. The Hedgehog (Hh) signaling pathway is essential for embryonic development and it is involved in the morphogenesis of organs like the lungs, brains, skeleton, and the gastrointestinal tract. In adults, it is involved in the self-renewal of stem cells and tissue regeneration [152,153,154]. Uncontrolled activation of this signaling pathway has been involved in the development of several cancers like basal cell carcinoma, glioblastomas, melanomas, and carcinomas of the ovary, prostate, breast, lung, and pancreas [155]. Thus, inhibition of this pathway shows promise as a cancer treatment [156,157,158]. The primary cilium is an MT-based organelle found in eukaryotic cells that acts as a communication hub to couple extracellular signals with intracellular responses [159,160]. The primary cilium plays an essential role in Hh signaling in mammals [161]. Some known drugs that affect the Hh pathway have also been shown to affect MT dynamics, leading to disruptions in the proper localization of the pathway components [162,163]. Increasing evidence supports a role for the primary cilium in cancer progression and development, making the cilium a candidate target for cancer treatment (reviewed in [164]).

### 5.3. MTAs and Cell Death by Apoptosis

Taxanes can induce mitotic catastrophe in cancer cells [165]; cell death via proteases, such as cathepsins [166]; and cell death through autophagy [167]. Furthermore, paclitaxel can also promote cell death by affecting the signaling pathways responsible for preventing the build-up of reactive oxygen species (ROS) [168]. However, as described above, taxanes are mostly known for their ability to trigger caspase-induced apoptosis [128,169]. In fact, the first studies of cell death triggered by paclitaxel revealed that the principal antitumor effect was through apoptosis rather than mitotic arrest [170]. Taxanes induce apoptosis through both the intrinsic and extrinsic pathways. The intrinsic pathway, also known as the mitochondrial pathway, is activated by intracellular signals after detecting cell damage while the extrinsic pathway is initiated by extracellular ligands binding to death receptor. The intrinsic pathway activates caspase 9 and the extrinsic pathway the caspase 8, but both of them converge in the activation of caspase 3 [171,172]. Taxanes activate the initiator caspases 8 and 9, and the executioner caspases 3, 6, and 7. In fact, the siRNA inactivation of caspase 3 increases the cell survival of taxane-treated cells [173]. Taxanes bind to the anti-apoptotic BCL2 family proteins and block their activity [174]. Treatment with taxanes also seems to downregulate protein levels of anti-apoptotic players like Bcl-2 and Bcl-xL, and upregulate pro-apoptotic ones like BAD and BAX [175,176]. In fact, MEFs lacking pro-apoptotic factors like BAX and BAK were resistant to apoptosis induced by taxanes [177,178].

### 5.4. MTAs and Effects on the Tumor Vascular Tissue

Tumor cells require oxygen and nutrients to survive, and tumor-infiltrating blood vessels transport these resources to the tumor cells. In addition, tumor blood vessels are also required for metastasis initiation. When the tumor vasculature is disturbed and cannot exert this function, tumor cells begin to die, and the tumor becomes necrotic. Thus, targeting tumor vasculature represents another pathway to treat cancer, reduce the tumor mass, and block the pathway to metastasis. There are two ways to target the tumor vasculature: by blocking the formation of new blood vessels (antiangiogenic effects) or by disrupting the already established vasculature (vasculature-disrupting agents). MTAs have both antiangiogenic and vascular-disrupting properties because they inhibit endothelial cell proliferation, migration, and morphology. Early studies from 1993 showed that the vinca alkaloids, vincristine and vinblastine, decrease the blood flow in tumors and induce necrosis of the tumor cells [179]. Interestingly, most of these effects are seen after treating cells with low concentrations of MTAs. These low concentrations do not elicit the typical cytotoxic effects, such as mitotic arrest and apoptosis, seen at high concentrations commensurate with high levels of MT polymer loss. Rather, these lower concentrations alter MT dynamics enough to affect signaling pathways and the proper formation of focal adhesions and adherents junctions required for cell–cell interactions with fewer overtly toxic effects [180].

#### 5.4.1. Antiangiogenic Effects

The formation of new blood vessels requires the proliferation and migration of endothelial cells. Dynamic MTs are essential for both functions, and any alteration of the MT’s dynamicity elicits cellular defects that ultimately lead to reduced proliferation, migration, and angiogenic capacity. For example, concentrations of paclitaxel that are found to be antiangiogenic increase MT dynamics in endothelial cells [181,182]. MTAs like docetaxel, epothilone B, and vinblastine prevent angiogenesis through the inhibition of the small GTPases Rac1 and Cdc42, proteins involved in cytoskeleton rearrangements required for cell motility and migration [183]. Taxane increases the levels of acetylated tubulin in HUVECs (human umbilical vein endothelial cells) with a correlative inhibition cell motility [184]. Interestingly, in this same study, the authors found that MTAs caused translocation to the nucleus of the transcription factor FOXO3, a negative regulator of cell motility and a positive regulator of cell cycle arrest, senescence, and cell death [185].

Focal adhesions are multiprotein assemblies that are essential for cell migration and facilitate the interaction between the extracellular matrix (ECM) and the cellular cytoskeleton. Focal adhesion formation and turnover is regulated by different extracellular factors, among them the vascular endothelial growth factor (VEGF). Laulimalide is a natural MTA that inhibits tubule formation and VEGF-induced cell migration of HUVECs [186]. The inhibition of these two processes occurred at substantially lower concentrations than that required to inhibit proliferation. At low concentrations, MTAs inhibit cell migration by reducing the number of peripheral MTs, affecting cell polarity formation, and disturbing the maintenance of the cell’s leading edge. Changes in MT dynamics reduce the total number of focal adhesions and disrupt its highly regulated turnover [187,188]. MTAs like paclitaxel can inhibit cell migration by impacting the repositioning of the microtubule-organizing center during migration [189].

HIF-1α (hypoxia-inducible factor 1-alpha) is a transcription factor that controls the transcription of VEGF [190,191], and its overexpression induces tumor angiogenesis and increases the survival of cancer cells [190,192,193]. MTAs can disrupt the transport of HIF-1α mRNA and releases it from polysomes, impeding its translation [140]. Moreover, HIF-1α is transported to the nucleus by the motor dynein, and taxane treatments that alter the MT cytoskeleton inhibit the transport of HIF-1α to the nucleus and impede its function [141]. MTAs like taxotere, epothilone B, discodermolide, vincristine, and colchicine have been shown to inhibit HIF-1α nuclear accumulation and activity by disrupting MT function [194]. Finally, 2-methoxyestradiol (2ME2) is a natural molecule derived from the hormone estradiol that also alters MT dynamics and has antitumor and antiangiogenic properties [195,196,197]. 2ME2 impairs HIF-1α function and therefore inhibits angiogenesis [194,198].

It has been shown that the vinca alkaloid vinorelbine has antiangiogenic effects in human endothelial cells [199]. MTAs like the taxanes downregulate the expression of Angiopoietin-1 (Ang-1), a protein required for vascular development and angiogenesis [200], and upregulate the expression and increased secretion of Thrombospondin 1 in the tumor microenvironment, a protein that is able to inhibit the formation of new blood vessels [201,202].

#### 5.4.2. Vasculature-Disrupting Effects

The antiangiogenic activity of some drugs is often ineffective as tumors find alternative mechanisms for angiogenesis activation. Therefore, a useful alternative is to target existing tumor vasculature using vascular-disrupting agents (VDAs). Interestingly, the tumor vasculature is different from the blood vessels in normal tissues, suggesting that it would be possible to specifically target the tumor vasculature while leaving the normal blood vessels intact. There are three classes of VDAs: MTAs, flavonoids, and vascular targeted drugs based on endothelial cell receptors [180,203,204,205,206,207].

The most highly studied MTAs used as VDAs are combretastatin molecules. Combretastatins are small molecules found in the bark of the *Combretum caffrum* and *Combretum leprosum* trees. They inhibit angiogenesis, cell proliferation, and cell migration (reviewed in [208]). The natural form known as Combretastatin A-4 (CA4) has low solubility in water, necessitating the synthesis of several water-soluble options: a phosphorylated form (CA4P) and a version with increased cytotoxic activity against tumor cells (CA1P) [209,210]. Combretastatins bind to the colchicine binding site of β-tubulin, and induce cell cycle arrest in G2/M, and cell death through apoptosis and mitotic catastrophe [211,212]. Killing the endothelial cells in blood vessels in this manner leads to vascular leakage, blockage of blood flow, poor transport of nutrients to cancer cells, and subsequent necrosis of the tumor [203,213,214]. Fortunately, combretastatins are more efficient at disturbing endothelial tumor cells than normal cells, but the exact molecular mechanisms behind this effect are not well-known [215,216,217]. One possibility is that the blood vessels newly formed around a tumor are still immature, unstable, and not properly formed [218]. Cells on new formed blood vessels rely more on the MT cytoskeleton to provide rigidity and shape to the vessel, while already mature vasculature relies more on the actin cytoskeleton. Thus, small changes in MT dynamics could affect the cell cytoskeleton and polarization and migration of new endothelial cells, which would contribute specifically to tumor vessel destruction. Combretastatins can also reduce the blood flow in normal blood vessels, but this reduction is temporary and the morphology and functionality of normal vessels remains intact [219].

Vinblastine and vincristine also possess anti-vascular properties, but they act at high concentrations, close or even higher than the maximum tolerated dose [220,221]. Plinabulin binds to the colchicine site and causes similar MT depolymerization and similar anti-vascular effects as other MTAs [222]. The tubulin polymerization inhibitor CDK-516 also has anti-vascular properties. It induces apoptosis, inhibits tumor growth in mice, and is especially effective in combination with other treatments like doxorubicin [223,224,225].

### 5.5. MTAs, Metastasis, and Cell Migration

One of the most dangerous characteristics of cancer cells is their ability to spread outside of the main tumor mass, travel through the body, and establish new tumors in other organs. This process, known as metastasis, is dependent on cell migration (reviewed in [226]). Initially cells lose their cell-to-cell adhesion capabilities and acquire a migratory and invasive phenotype. This is known as the epithelial to mesenchymal transition. Following this transition, the new cells invade the stroma, enter in the blood and lymphatic vessels, and begin circulating throughout the body. Finally, the metastatic cells leave the vessels in a process known as extravasation, and colonize new tissues. All these steps during the metastatic process require a functional MT and actin cytoskeleton for proper cell movement, changes in cell morphology, and cell migration.

MTs are essential for cell polarization and cell migration [227]. For example, T cells have the ability to polarize their actin and MT cytoskeleton to be able to orient themselves toward the antigen-presenting cells and to migrate [228]. The MSA Zampanolide inhibits cell migration [229], and Epothilone B is able to inhibit cell migration of glioblastoma cells without overtly affecting MT dynamic criteria, such as the accumulation of EB1 at microtubule plus ends or the MT growth rate [230]. However, most cell migration studies have been done in traditional 2D tissue culture systems. New cell migration studies in organoids and 3D cell culture systems seem to indicate that MTAs more profoundly affect the cell migration of cells in 3D systems than in 2D cultures. For example, paclitaxel is 100-fold more effective at blocking migration in a 3D matrix than on a 2D matrix [231]. A good review to understand the role of MTs in 3D cell motility was published in 2017 [232]. Notably, the concentrations of MTAs required to affect cell migration are much lower than the concentrations required to trigger a cytotoxic effect. This is probably because low concentrations may be capable of significantly altering the behavior of the MT network close to the cell membrane without triggering apoptosis [233].

As in the case of the tumor vasculature, focal adhesion turnover is also essential during metastasis. Proper MT dynamics is required for the focal adhesions’ assembly-disassembly cycle necessary for cell migration. Disturbing this cycle with MTAs like nocodazole inhibits cell migration required for metastasis [188,234].

In cancer cells, invadopodia are small evaginations of the cell membrane that are required to contact the extracellular matrix (ECM) and start the invasion of adjacent tissues. These invadopodia grow to form long membrane protrusions that promote invasion. In a 3D model for cell invasion, where cells were placed in contact with native collagen type-I matrix overlaid with a thin basement membrane equivalent, paclitaxel was able to inhibit the formation of long membrane protrusions that are required for efficient invasion [235]. Interestingly, paclitaxel did not affect the initial formation of invadopodia but instead prevented the recruitment of MTs to complete the assembly of long and functionally effective membrane protrusions [236].

## 6. MTAs and Drug Resistance

MTAs have been used to treat different pathologies for decades. However, treatment with MTAs for long periods of time can trigger resistance to therapy.

The most common method of drug resistance is to expel the therapeutic compounds out of the cell through cellular transporters. The ATP-binding cassette (ABC) transporters is a family of transmembrane proteins that transport molecules across the cell membrane. At least 11 ABC transporters are involved in multidrug resistance (MDR) development to different treatments. Examples of these transporters are the P-glycoprotein 1 transporter (P-GP/ABCB1), the breast cancer resistance protein (BCRP/ABCG2), and the multidrug resistance-associated proteins (MRPs/ABCCs) [237]. The P-glycoprotein 1 transporter (ABCB1) is the most well-studied, the most prevalent drug resistance system, and the most important transporter from the clinical point of view [238,239,240]. This transporter can move taxanes out of cancer cells, rendering taxane therapy ineffective over time [241,242,243]. Another taxane transporter is the member of the same family ABCC3 [244]. One factor that contributes to the cell’s ability to expel these drugs is based on the fact that most MTA-tubulin binding events are transitory and reversible. There are some MSAs that are able to bind in a covalent manner to the tubulin, making these compounds very useful to avoid drug resistance [245,246].

Another method to generate drug resistance is the expression of different isotypes of β-tubulin to desensitize cells to MTAs [247]. Cancer cells favor the expression of βIII-tubulin, which interferes with the suppression of dynamic instability by taxanes [248,249,250,251]. In fact, inhibiting βIII-tubulin by siRNA renders cells more sensitive to taxanes and triggers cell death by apoptosis [252,253]. βIII-tubulin expression may influence MT dynamics [254] or, alternatively, may influence signaling pathways important for tumorigenesis [255]. For these reasons, new MTAs and combination therapies are being designed and tested to overcome βIII-tubulin-dependent drug resistance [256,257,258,259].

Finally, we would like to mention another shortcoming of most MTAs. Similar to the limitations described above in treating neurodegenerative diseases of the brain, many MTAs do not cross the BBB. This limits their ability to target brain cancers, such as gliomas. Accordingly, there is considerable interest in adapting Epothilone derivatives [260] and in developing novel MTAs [261,262] that do cross the BBB to target these devastating cancers.

## 7. Conclusions

Housekeeping proteins are essential proteins that are involved in the basic functioning of a cell. Tubulin is, therefore, a housekeeping protein, as MTs participate in a wide variety of essential cellular activities including but not limited to cell division. This presents both opportunities and challenges for the use of MTAs in the treatment of abnormal cell growth associated with cancer and other cell physiological pathologies. Because MTs are broadly essential for cellular functions, MTAs exhibit well-documented toxic effects against normal tissue. However, proper MT function is exquisitely dependent on MT dynamics and turnover, the key parameters of which are often tissue, cell, or activity specific. This has contributed to the surprising therapeutic efficacy of many MTAs. For example, even mild modulation of MT dynamics can, often fortuitously, direct the efficacy of these drugs toward specific tissues, unique signaling processes, or even exclusively to cancerous tissue. More research into how dynamic MTs impact broad cellular activities will enable greater effective utilization of MTAs against human disease.
